# Prognostic impact of tumor length in esophageal Cancer: a systematic review and Meta-analysis

**DOI:** 10.1186/s12885-021-08728-1

**Published:** 2021-09-03

**Authors:** Zhao Yang Wang, Yuan Zhu Jiang, Wen Xiao, Xian Biao Xue, Xiang Wei Zhang, Lin Zhang

**Affiliations:** 1 Department of Thoracic Surgery, Shandong Provincial Hospital Affiliated to Shandong First Medical University, 324 Jing wu Road, Jinan, 250021 Shandong China; 2 Department of Thoracic Surgery, Tangdu Hospital, The Fourth Military Medical University, 1 Xinsi Road, Xi’an, 710038 China; 3 Department of Thoracic Surgery, Juye County People’s Hospital, Ju ye, China

**Keywords:** Esophageal cancer, Tumor length, Prognosis, meta-analysis, Systematic review

## Abstract

**Background:**

In clinical studies, it has been observed that esophageal cancer (EC) patient prognosis can be very different even for those patients with tumors of the same TNM stage. Tumor length has been analysed as a possible independent prognostic factor in many studies, but no unanimous conclusion has been reached. Therefore, this review used a meta-analysis to evaluate the association between tumor length and prognosis in EC patients.

**Methods:**

A systematic search for relevant articles was performed in PubMed, Web of Science, and Embase. Hazard ratios (HRs) and 95% confidence intervals (CIs) were used as effective measures to estimate the correlation between tumor length and prognosis, including overall survival, disease-free survival, progression-free survival, disease-specific survival, and cancer-specific survival. STATA 15.0 software was used to perform the meta-analysis and the data synthesis.

**Results:**

Finally, 41 articles with 28,973 patients were included in our study. The comprehensive statistical results showed that long tumors are an independent prognostic parameter associated with poor overall survival (OS) (HR = 1.30; 95% CI: 1.21–1.40, *p* < .001) and disease-free survival (DFS) (HR = 1.38; 95% CI: 1.18–1.61, *p* < .001) in EC patients. Subgroup analyses also suggested a significant correlation between long tumors and poor OS. Sensitivity analysis and publication bias evaluation confirmed the reliability and stability of the results. Similar results were obtained in the analyses of progression-free survival (PFS), disease-specific survival (DSS), and cancer-specific survival (CSS).

**Conclusion:**

The results of this meta-analysis showed that long tumors were related to poor OS, DFS, PFS, DSS and CSS in EC patients. Tumor length might be an important predictor of prognosis in EC patients, and it can be used as an independent staging index. Further well-designed and large-scale prospective clinical studies are needed to confirm these findings.

## Background

Esophageal cancer (EC) is one of the most common and fatal cancers of the digestive system in the world. In the United States, more than 18,000 people are diagnosed with EC every year, and more than 15,000 people die of EC. Most patients die within 1 year of diagnosis [1]. The main methods to improve the prognosis of EC are surgery, chemotherapy and radiotherapy [2, 3]. Although there are established radiotherapy and chemotherapy regimens, surgical resection is still the only possible cure for this disease. Complete surgical resection and radical lymph node dissection can provide accurate pTNM staging, which has important guiding significance for predicting prognosis and determining treatment options after surgery. An effective and reasonable EC staging system is important for the selection of treatment options and the prediction of long-term survival [4].

The current classification criteria for EC patients include histological grading, primary tumor (T), lymph node metastasis (N), and metastasis (M). Tumor length was not included as a prognostic indicator [5]. In recent years, many studies have suggested that tumor length is an independent prognostic factor. Gaur et al. [6] and Feng et al. [7] analysed 296 EC patients who underwent surgical resection and found that tumor length was a risk factor for long-term survival in both SCC and AC patients. Shimada et al. [8] and Song et al. [9] retrospectively reviewed 575 patients with EC who underwent curative resection and found that tumor length had a similar negative effect on patients with different pTNM stages. Our early studies showed that tumor length was an important prognostic factor for OS and cancer-specific survival (CSS) [10, 11]. However, the prognostic role of tumor length in EC is still controversial. The results of Khan et al. [12] indicated that tumor length was not correlated with prognosis in N0 EC patients. Therefore, the purpose of this study was to evaluate the prognostic value of tumor length for the survival of EC patients by performing a meta-analysis.

## Methods

### Data sources and search strategy

All data included in this study are publicly available in PubMed, Embase, and Web of Science. Our meta-analysis was based on the Preferred Reporting Items for Systematic Reviews and Meta-Analyses (PRISMA) 2009 guidelines [13]. This systematic review and meta-analysis has been registered on PROSPERO with registration number: CRD CRD42018106851. The PubMed, Embase, and Web of Science databases were searched to identify relevant articles for analysis from 1994 until December 2019. All keywords were searched through MeSH. The full text of all included articles must be available, and the reference lists of each identified publication were reviewed for potential studies to avoid omission. The search strategy for PubMed was as follows: (esophageal carcinoma [Title/Abstract] OR esophageal cancer [Title/Abstract] OR “EC” [Title/Abstract] OR esophageal adenocarcinoma [Title/Abstract] OR esophageal malignancy [Title/Abstract] OR esophageal neoplasm [Title/Abstract]) AND (tumor length [Title/Abstract] OR tumor size [Title/Abstract]) AND (survival [Title/Abstract] OR prognosis [Title/Abstract] OR prognostic [Title/Abstract] OR outcome [Title/Abstract]).

### Inclusion and exclusion criteria

Articles were included if they met the following criteria:

1. Patients with EC were histopathologically confirmed, including SCC and AC;
2. Studies explored the relationship between the tumor length and prognosis of EC;
3. The hazard ratios (HRs) and 95% CIs between tumor length and survival outcomes were reported.

### Articles were excluded if they met the following criteria

1. They were abstracts, case reports, reviews, letters, or editorials;
2. Studies were not published in English;
3. Studies had overlapping or repeating data;
4. Studies did not present the cut-off value for tumor length.

### Data extraction and quality assessment

Two researchers (ZW and YJ) independently reviewed articles that met the inclusion criteria. Any disagreements were resolved by discussion or arbitrator (WX). The HRs and 95% CIs were extracted and pooled according to different prognoses (OS or DFS/PFS/DSS/CSS) to analyse the relationship between tumor length and prognosis. All HRs were extracted from the multivariate analysis. The data were obtained directly from the included articles or from simple indirect data calculations [14]. In addition, other parameters were extracted in a uniform format, including author, year of publication, patient source, total number of patients, patient age, follow-up time, treatment strategy, LN metastasis, histology, tumor location, TNM stage, cut-off value of tumor length and survival data. The quality of the included studies was assessed through the Newcastle-Ottawa Quality Assessment Scale (NOS), which consists of 3 parts: selection (0–4 points), comparability (0–2 points), and outcome assessment (0–3 points). The maximum score was 9 points, and studies with no less than 6 points were defined as high-quality studies [15].

### Statistical analysis

All statistical analyses were performed using STATA software version 15.1 (STATA, College Station, TX). The pooled HRs and 95% CIs were used to assess the prognostic role of tumor length in EC patients. A pooled HR > 1 indicated a worse prognosis in EC patients with long tumors. *P* < .05 for the Q test or *I*^2^ > 50% for the I^2^ test indicated significant heterogeneity in the literature, and the random-effects model (DerSimonian-Laird method) was adopted [16]; otherwise, the fixed-effects model (Mantel-Haenszel method) was used [17]. Subgroup analyses were performed based on patient source, histology, treatment, sample size, median age, cut-off value, percentage of patients with LN metastasis, percentage of patients with TNM stage III/IV classification and HR analysis method to explore the potential sources of heterogeneity and assess the stability of our aggregated results. Sensitivity analyses were performed by sequentially removing individual studies to test the robustness of the pooled results of these studies. Publication bias was assessed by Begg’s funnel plot and Egger’s linear regression test [18, 19]. The trim-and-fill method was used to evaluate the impact of publication bias on the pooled results in the presence of significant publication bias. *P* < .05 was considered statistically significant.

## Results

### The characteristics of the included studies

**Fig. 1:**
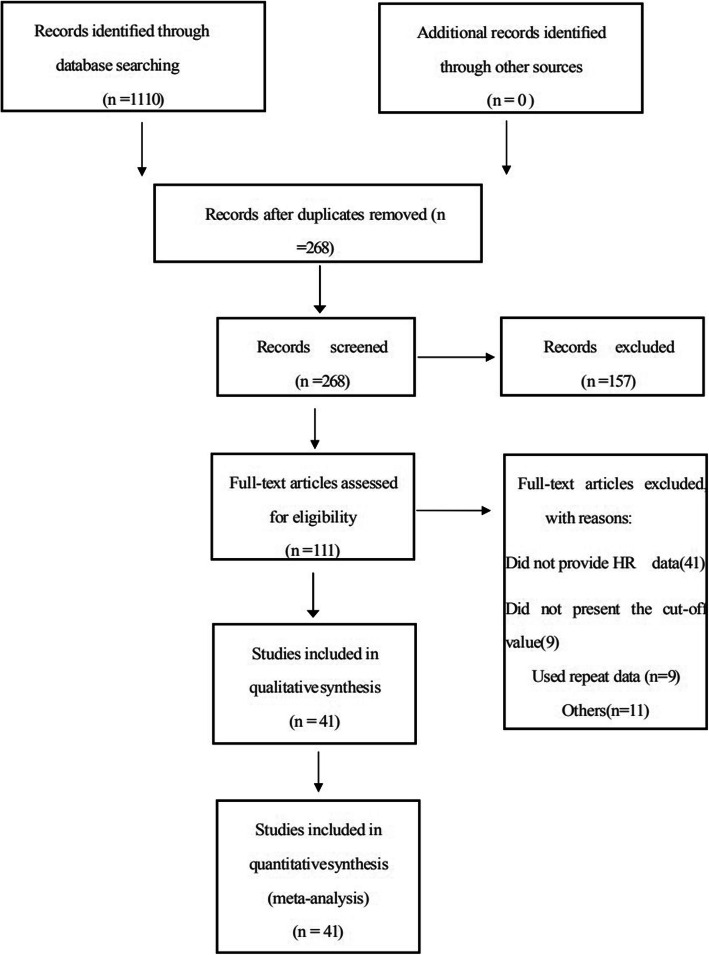
Flow chart of the literature selection process

Based on the search strategy mentioned above, a total of 1110 articles were searched from PubMed, Embase and Web of Science. Finally, 41 studies published between 1994 and 2019 with a total of 28,973 patients were included in our meta-analysis according to the inclusion and exclusion criteria [6–10, 20–55]. The specific literature selection process is shown in Fig. 1.

Table 1 summarizes the characteristics of all the studies included in this meta-analysis. All of the included studies were retrospective. Among these studies, 23 studies were from China [7, 9, 10, 30–49], 7 studies were from the USA [6, 50–55], 7 studies were from Japan [8, 24–29], and 1 study each was from Italy [22], Australia [20], the Netherlands [21], and Turkey [23]; the participants in 39 studies were patients with SCC. Three of the 41 studies included patients who received chemoradiotherapy only [26, 48, 53], and the remaining 38 studies included patients who underwent surgical resection with or without chemoradiotherapy. Among the 41 studies, 35 studies reported the relationship between the tumor length of EC and OS, 10 studies reported disease-free survival (DFS), 2 reported progression-free survival (PFS) [48, 53], 1 reported cancer-specific survival (CSS) [55] and 1 reported disease-specific survival (DSS) [20]. The cut-off value (cm) applied in all studies was between 1.5 and 6. Eighteen studies used a tumor length cut-off value of ≥5, and 23 studies used a tumor length cut-off value of < 5. All the tumor length values in the Surg and Surg^2^ groups were measured from postoperative pathological specimens, and the data from the chemoradiotherapy (CRT) group and Surg^3^ group were measured by endoscopy or computed tomography (CT) before treatment. The Surg^1^ group did not clearly describe the timing of measurement according to the different treatments. Because of the irregular shapes of the tumors, the actual size of them is difficult to measure. Most studies only analyzed the relationship between tumor length and patient prognosis, but did not discuss the effect of width. So, we default that the length of the tumor is proportional to its size, and combined the results of included studies on this basis. Two studies reported only relative risk (RR) data [41, 42], and we used RR to replace HR when pooling the data.

**Table 1:** Main characteristics of all included studies

Author	Publication year	Patient source	No. of patients	Age (years) (median and range)	Follow-up (months) (median and range)	Treatment	LN metastasis (% of total)	histology	Tumor location			TNM stage	III/IV (% of total)	Cut-off value (cm)	Survival analysis	HR reported	NOS
									Upper	Middle	Lower						
Tachibana, M.	1999	Japan	65	65	NR	Surg^2^	30.77	SCC	6	59		T1–2N±	NR	4	OS	MV	7
Shimada, H.	2004	Japan	374	65	NR	Surg^2^	65.78	SCC	98	276		I-IV	77.0	5	OS	MV	6
Barbour, A. P.	2008	Australia	131	61(30–78)	61	Surg^3^	51.15	SCC, AC	4	28	85	0-III	32.8	6	DSS	MV	8
Yendamuri, S.	2009	US	209	64 (33–84)	NR	Surg^2^	45.45	SCC, AC	3	27	179	I-III	34.9	3	OS	UV/MV	7
Shitara, K.	2010	Japan	363	63	67.2 (25.2–94.8)	Surg^1^	NR	SCC	NR			I-IV	56.2	5	OS	UV/MV	7
Heijl, M. V.	2010	The Netherlands	199	64 (35–78)	60+	Surg^2^	72.36	SCC, AC	NR			NR	NR	4	OS/DFS	UV/MV	8
Gaur, P.	2011	US	164	65 (26–84)	NR	Surg^2^	14.63	AC	0	8	156	NR	NR	2	OS	UV/MV	8
Lu, C. L.	2011	China	127	59 (39–77)	NR	Surg^2^	32.28	SCC	NR			I-III	30.7	2	OS/DFS	MV (RR)	7
Wang, B. Y.	2011	China	582	65.4 (30–88)	31.9	Surg^2^	54.64	SCC	77	340	165	T1-4N0–3	NR	3	OS	MV	7
Yamamoto, S.	2011	Japan	170	64	NR	Surg^1^	NR	SCC	18	107	45	I	NR	5	OS	MV	6
Song, Z. B.	2012	China	201	59 (31–78)	52 (30–136)	Surg	NR	SCC	12	102	87	T_1_-_2_	NR	3	OS	MV	7
Chen, J.	2012	China	945	NR	NR	Surg^2^	100	SCC	146	712	87	T1-4N+	NR	5	OS	UV/MV	7
Feng, J. F.	2013	China	132	73.6 ± 2.6	NR	Surg^2^	56.11	SCC	6	55	71	T1-4N0–3	NR	4	OS	UV/MV	6
Matsumoto, S.	2013	Japan	32	63	NR	Surg^2^	42.86	SCC	3	20	6	I-III	71.9	6	OS	MV	7
Zeybek, A.	2013	Turkey	116	60.0 (33–75)	39.7	Surg^2^	65.52	SCC, AC	10	36	70	II-III	64.7	3	DFS	MV	6
Chen, L. J.	2014	China	103	58	NR	Surg^2^	47.57	SCC	NR			I-IV	33.0	3.5	OS	UV/MV	7
Shridhar, R.	2014	US	154	65	NR	Surg^1^	84.42	AC	0	5	80	I-IV	68.8	5	OS	MV	6
Freilich, J.	2015	US	232	64.4 ± 11.3	25.9 (2.5–124.2)	Surg^1^	83.19	SCC, AC	8	17	118	I-IV	72.8	5	OS	MV	7
Hulshoff, J. B.	2015	US	105	64 (57–69)	29 (15.–56.0)	Surg^1^	73.33	SCC, AC	0	12	49	NR	NR	5	DFS	UV/MV	6
Ma, M. Q.	2015	China	362	54.5	84 (6–144)	Surg^2^	12.18	SCC	28	243	100	I-III	25.1	4	OS	MV (RR)	8
Miao, L. S.	2015	China	1342	59.5 ± 7.9	30	Surg^2^	45.98	SCC, AC	NR			I-IV	41.1	4	OS	UV/MV	7
Hirahara, N.	2016	Japan	147	65.7	NR	Surg^1^	59.86	SCC	8	65	52	I-III	37.4	3	CSS	UV/MV	7
Hwang, J. Y.	2016	China	294	55	20.4	Surg^2^	58.84	SCC	51	114	129	I-IV	49.3	3.2	OS	UV/MV	7
Jia, W.	2016	China	83	NR	NR	Surg^2^	56.63	SCC	8	44	31	I-III	43.4	5	OS/DFS	MV	6
Ma, Q. L.	2016	China	725	58 (32–80)	NR	Surg^2^	46.48	SCC	NR			I-III	38.2	5	OS	UV/MV	7
Sakanaka	2016	Japan	144	67 (41–85)	48 (13–88)	CRT	74.31	SCC	33	79	32	I-IV	63.9	4	OS	MV	7
Valmasoni, M.	2016	Italy	357	62 ± 9.3	NR	Surg^2^	50.98	SCC	90	147	120	I-III	47.6	3	OS	MV	7
Valmasoni, M.	2016	Italy	305	63 ± 11.2	NR	Surg^2^	66.23	AC	0	6	299	I-III	58.4	3	OS	MV	7
Wu, J.	2016	China	1435	58.3	24 (1–128)	Surg^2^	53.24	SCC	33	691	711	T1-4N0–3M0–1	NR	4	OS	UV/MV	7
Duan, J.	2016	China	328	61	44.9 (3.4–107.6)	Surg^2^	42.4	SCC	24	202	102	I-III	57.6	4.2	OS/DFS	UV/MV	8
Gao, S. H.	2016	China	126	NR	NR	Surg^1^	85	SCC	NR			I-III	54.8	4	OS/DFS	MV	6
Li, S. P.	2016	China	100	59.2 ± 10.3	NR	Surg^1^	55	SCC	12	41	37	I-III	54.0	4	OS/DFS	MV	6
Tian, R.	2016	China	442	60 (20–88)	NR	Surg^2^	47.5	SCC	39	277	126	I-III	43.7	5	OS/DFS	UV/MV	7
Xi, M.	2017	US	496	67 (20–92)	24.2 (2.8–155.9)	CRT	70.97	SCC, AC	138		358	I-III	67.9	5	PFS	MV	7
Zeng, Y.	2017	US	1242	NR	NR	Surg	NR	SCC, AC	39	136	913	NR	NR	1.5	OS/CSS	MV	7
Li, J.	2017	China	294	58 (38–70)	26	Surg^2^	67.3	SCC	6	139	149	T1-4N±	NR	5	OS	MV	7
Yang, Y. S.	2017	China	508	59	37.5 (1–105)	Surg^2^	40.9	SCC	78	260	170	T1-4N±	NR	4	OS	UV/MV	7
Zhang, X. W.	2017	China	498	59 (38–81)	47.2 (6.–64.5)	Surg^2^	41	SCC	NR			T1-4N±	NR	3	OS	UV/MV	7
Bai, G.	2018	China	80	54.7 ± 12.6	28 (9–62)	Surg^2^	52.5	SCC	24	36	20	T1-4N±	NR	5	OS/DFS	MV	6
Cheng, Y. F.	2018	China	14,394	NR	NR	Surg^1^	73.5	SCC	2897	5028	3213	0-IV	71.5	5	OS	MV	6
Xu, H. Y.	2018	China	751	65	56.6 (25.2–112.5)	CRT	78.96	SCC + Others	157	420	128	T1-4N0–3	NR	5	PFS	UV/MV	7
Gu, L.	2019	China	116	NR	NR	Surg^1^	85.34	SCC	NR			I-IV	55.2	5	OS	UV/MV	6

*No*. number, *HR* hazard ratio, “M” means the HR came from multivariate analysis, “U” means the HR came from univariate analysis, *NOS* Newcastle –Ottawa Quality Assessment Scale, *R* retrospective, *SCC* squamous cell carcinoma, *AC* adenocarcinoma, *OS* overall survival, *PFS* progression-free survival, *DFS* disease-free survival, *DSS* disease-specific survival, *CSS* cancer-specific survival, *NR* not reported, *CRT* chemoradiotherapy, Surg^1^:±Neo CRT/±Surg/±Adj CRT; Surg^2^: Surg±Adj CRT; Surg^3^: Neo CRT + Surg

### Tumor length and OS in EC

**Fig. 2:**
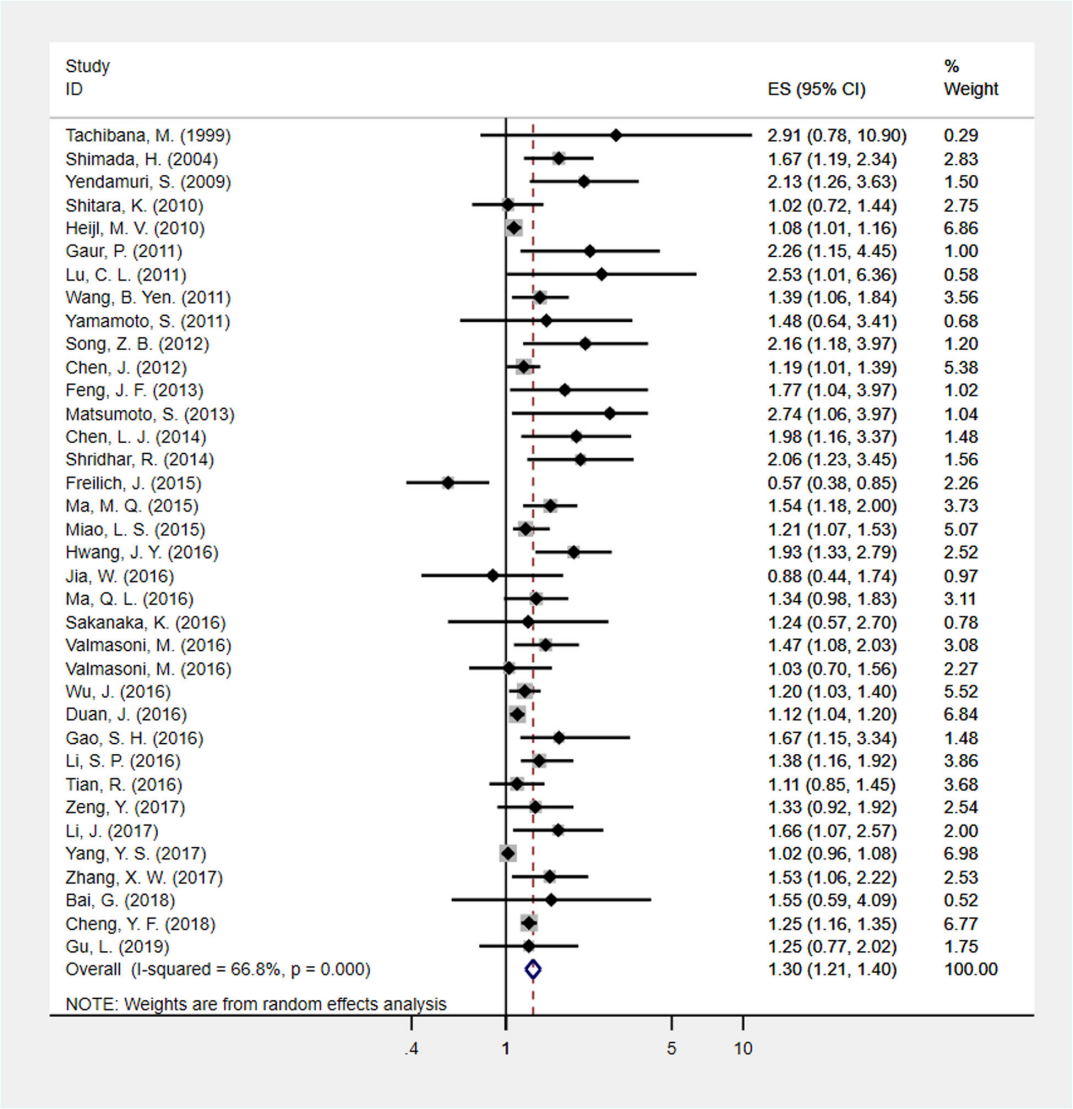
Meta-analysis of the relationship between tumor length and OS in EC patients. EC = esophageal cancer, OS = overall survival

A total of 35 studies evaluated the relationship between tumor length and OS in EC, and because of the significant heterogeneity among the included studies (*I*^2^ = 66.8%; *ph* < .001), the pooled HR and 95% CI were calculated by a random-effects model. The pooled HR of 1.301 (95% CI: 1.210–1.399) suggests that long tumors are associated with poor OS (Fig. 2).

### Tumor length and DFS in EC

**Fig. 3:**
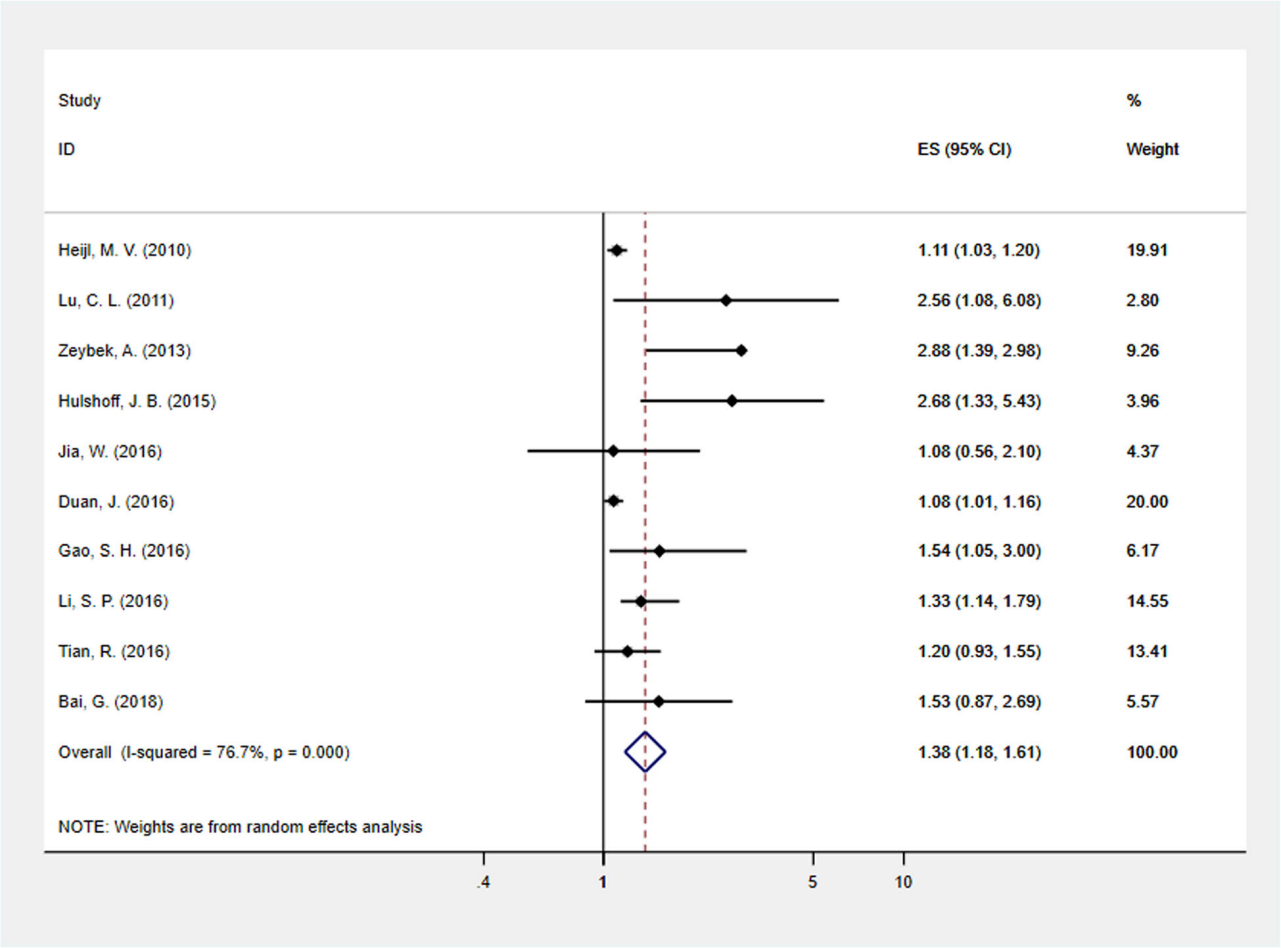
Forest plot to evaluate the prognostic significance of tumor length for the DFS of EC patients. HR = hazard ratio, DFS = disease-free survival, EC = esophageal cancer

Ten studies provided the HR and 95% CI of tumor length in association with DFS. The pooled data showed that long tumors were associated with poor DFS (HR = 1.378; 95% CI: 1.179–1.609, *p* < .001). Due to obvious heterogeneity (*I*^2^ = 76.7%, *ph* < .001), the random-effects model was used to calculate the pooled HR and its 95% CI (Fig. 3).

### Tumor length and PFS in EC

No significant heterogeneity was found among the studies that evaluated PFS, so a fixed-effects model was used to calculate the pooled HR and 95% CI (HR = 1.161; 95% CI: 1.008–1.337, *p* < .005). The data suggest that long tumors were associated with shorter PFS.

### Tumor length and CSS/DSS in EC

Two studies reported preoperative tumor length and CSS/DSS data for EC. There was no significant heterogeneity (*I*^2^ = 0.0%, *ph* = .551). The pooled HR and 95% CI (HR = 1.856; 95% CI: 1.173–2.937, *p* < .001) indicated that long tumors were also related to poor CSS/DSS.

### Subgroup analysis

To analyse the impact of heterogeneity on the results of this study, we performed subgroup analyses on the extracted data. Subgroup analyses by patient source (China and others), histology (SCC, AC and mixed), treatment (Surg^2^ and others), median age (< 60 and ≥ 60), cut-off value (< 5 and ≥ 5), sample size (< 200 and ≥ 200), percentage of patients with LN metastasis (< 50% and ≥ 50%), percentage of patients with TNM stage III/IV classification (< 50% and ≥ 50%) and HR analysis method (MV and UV) were performed to explore the potential sources of heterogeneity for the pooled OS results. Because the cut-off values of tumor length were different among the included studies, we performed subgroup analysis according to different cut-off values. Because 40% of articles chose 5 cm as the cut-off value for tumor length, we used this cut-off value as the boundary. For cut-off value ≥5, the pooled HR was 1.259 (95% CI: 1.096–1.446, *I*^2^ = 59.4%, *ph* = .002). For cut-off value < 5, the pooled HR was 1.322 (95% CI: 1.210–1.443, *I*^2^ = 67.9%, *ph* < .001). This suggests that we may be able to define 5 cm as a standard cut-off value and recommend it to other researchers to reduce heterogeneity between different studies. The results of subgroup analyses showed that the different classification methods had no obvious influence on HR (Table 2). In almost all subgroups, long tumors were significantly related to poor OS, which showed that our pooled HR result for OS was stable and reliable. Considering the limited research on tumor length and DFS, PFS, DSS, and CSS, no other subgroup analysis was performed.

**Table 2:** Subgroup analyses reflecting the association between tumor length and OS in EC patients

		Random-effects model		Fixed-effects model			
Subgroup	No. of studies	HR (95% CI)	*P*	HR (95% CI)	*P*	I^2^ (%)	Ph
Overall	35	**1.301 (1.210–1.399)**	<.001	1.151 (1.117–1.185)	<.001	66.8%	<.001
Patient source							
China	23	**1.290 (1.191–1.397)**	<.001	1.155 (1.117–1.195)	<.001	64.6%	<.001
Japan	6	1.540 (1.108–2.142)	.010	**1.446 (1.174–1.781)**	<.001	47.0%	.093
US	5	**1.457 (0.851–2.495)**	.170	1.288 (1.046–1.586)	.017	84.1%	<.001
The Netherlands	1	1.080 (1.008–1.157)	.029	1.080 (1.008–1.157)	.029	–	–
Italy	1	1.258 (0.890–1.778)	.193	1.283 (1.001–1.644)	.049	–	–
Histology							
SCC	28	**1.332 (1.227–1.445)**	<.001	1.163 (1.125–1.203)	<.001	64.0%	<.001
AC	3	**1.616 (0.950–2.748)**	.077	1.469 (1.103–1.957)	.009	67.9%	.044
Mixed	5	**1.128 (0.895–1.421)**	.307	1.095 (1.029–1.166)	.004	78.5%	.001
Treatment							
Surg^2^	24	**1.311 (1.207–1.423)**	<.001	1.127 (1.091–1.165)	<.001	67.3%	<.001
Others	11	**1.270 (1.066–1.514)**	.007	1.250 (1.170–1.336)	<.001	59.5%	.006
Sample size							
≤ 200	16	**1.458 (1.245–1.707)**	<.001	1.163 (1.098–1.233)	<.001	56.3%	.003
> 200	19	**1.264 (1.157–1.380)**	<.001	1.146 (1.108–1.186)	<.001	73.3%	<.001
Median age							
< 60	9	**1.422 (1.228–1.647)**	<.001	1.124 (1.071–1.179)	<.001	74.5%	<.001
≥ 60	14	**1.281 (1.132–1.450)**	<.001	1.133 (1.083–1.185)	<.001	67.5%	<.001
Cut-off value							
< 5 cm	21	**1.322 (1.210–1.443)**	<.001	1.123 (1.086–1.162)	<.001	67.9%	<.001
≥ 5 cm	14	**1.259 (1.096–1.446)**	.001	1.241 (1.169–1.317)	<.001	59.4%	.002
LN metastasis (% of total)							
< 50%	13	**1.351 (1.189–1.537)**	<.001	1.106 (1.061–1.153)	<.001	73.9%	<.001
≥ 50%	18	**1.281 (1.162–1.413)**	<.001	1.194 (1.144–1.246)	<.001	62.3%	<.001
TNM stage III/IV (% of total)							
< 50%	12	1.441 (1.240–1.676)	<.001	**1.377 (1.245–1.524)**	<.001	44.5%	.062
≥ 50%	14	**1.243 (1.088–1.421)**	.001	1.190 (1.134–1.249)	<.001	69.5%	<.001
HR estimate							
UV	16	**1.676 (1.469–1.914)**	<.001	1.218 (1.180–1.256)	<.001	91.3%	<.001
MV	35	**1.301 (1.210–1.399)**	<.001	1.151 (1.117–1.185)	<.001	66.8%	<.001

*HR* hazard ratio, *CI* confidence interval, *Ph P* value of Q test for heterogeneity test, *SCC* squamous cell carcinoma, *AC* adenocarcinoma; *UVA* univariate analysis, *MVA* multivariate analysis, *LN* lymph node, *Surg*^*2*^ Surg±Adj CRT

### Sensitivity analyses

**Fig. 4:**
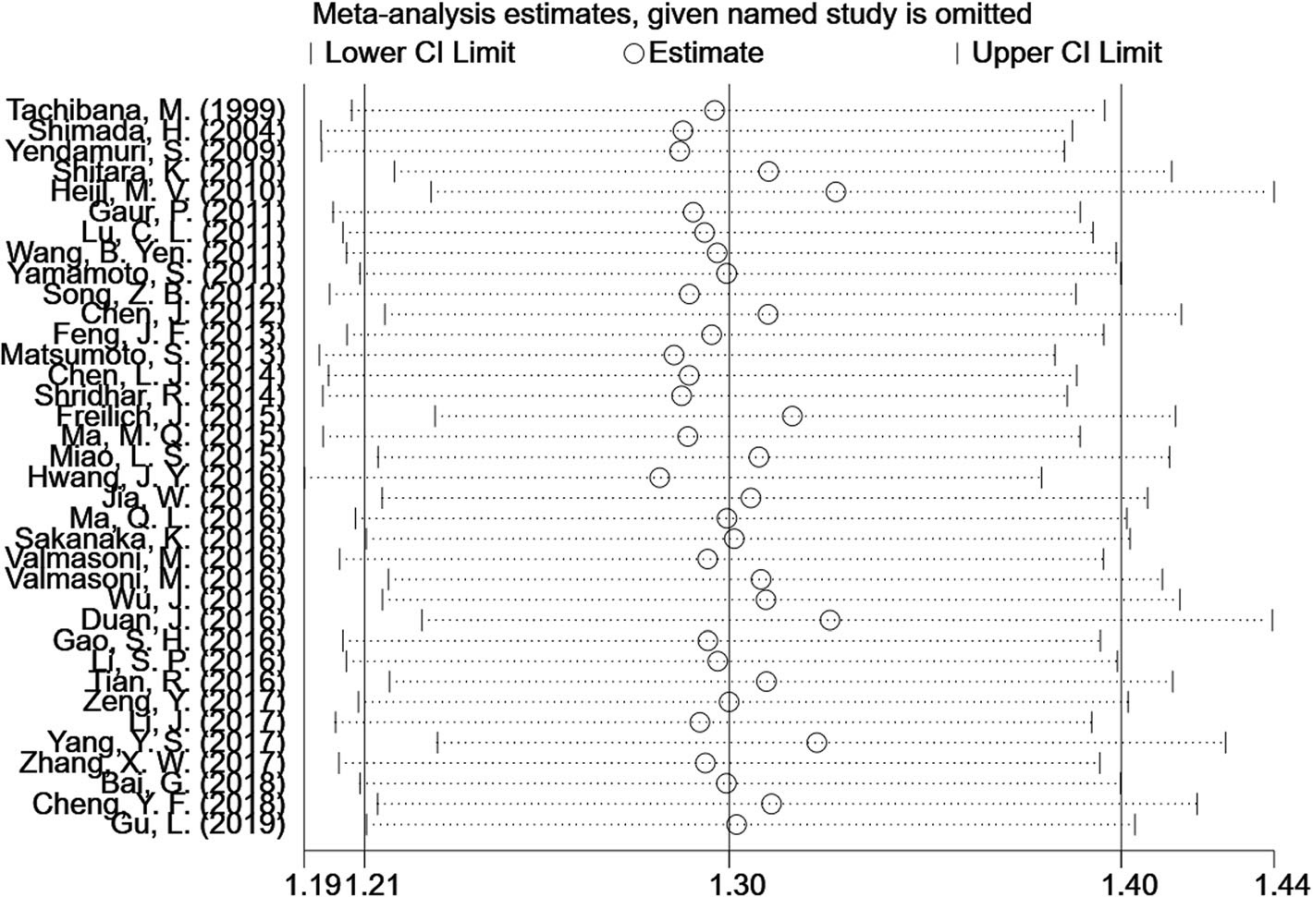
Sensitivity analysis of tumor length and OS in EC patients. EC = esophageal cancer, OS = overall survival

**Fig. 5:**
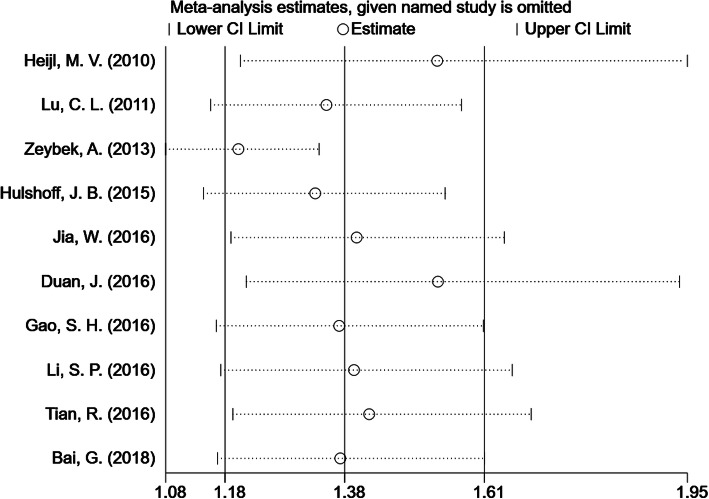
Sensitivity analysis of tumor length and DFS in EC patients. EC = esophageal cancer, DFS = disease-free survival

After omitting each individual study for the sensitivity analysis, the results showed that when excluding any study, the pooled HRs did not change substantially, indicating that our results were stable (Figs. 4, 5).

### Publication bias

**Fig. 6:**
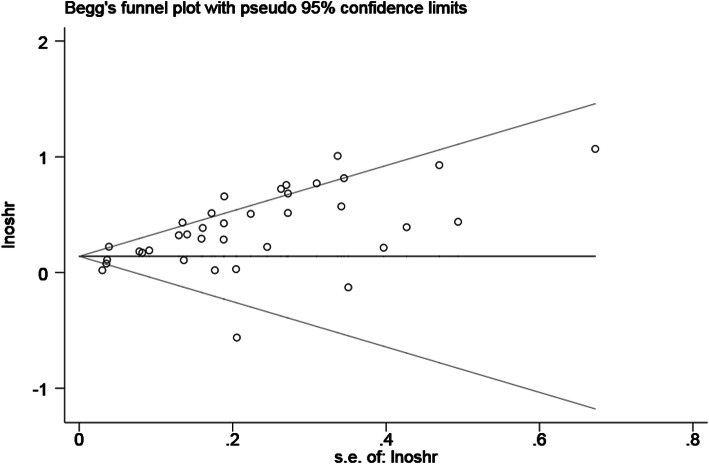
Begg’s test funnel plot of the publication bias of tumor length and OS. OS = overall survival

**Fig. 7:**
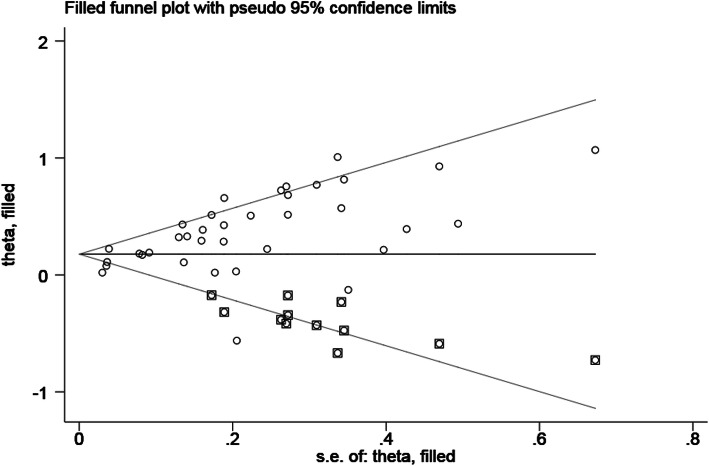
Funnel plot after using the trim-and-fill method to adjust for OS. OS = overall survival

Publication bias was evaluated by Begg’s funnel plot and Egger’s linear regression test. Considering the limited number of studies on the relationship between tumor length and DFS, PFS, DSS or CSS, we analysed the publication bias of tumor length and OS (Pr > |z| =0.127 for Begg’s test and *p* < .01 for Egger’s test) (Fig. 6). Therefore, we conducted a further trim-and-fill analysis (Fig. 7). The adjusted result (HR = 1.195, 95% CI: 1.111–1.284) was similar to our previous pooled result. This result showed that despite the presence of publication bias, it did not substantially affect the results of our study.

## Discussion

EC is one of the most lethal malignant tumors in the world, with a 5-year survival rate of less than approximately 20% [1]. With the development of clinical treatment technology, many comprehensive treatment methods, including surgery, chemotherapy and radiotherapy, have been used in EC patients. Combined with the extensive development of minimally invasive McKeown esophagectomy (MIE-McKeown), we can obtain accurate pTNM staging information for each EC patient and use the system to guide the choice of further treatment and the prediction of long-term survival [4]. These assessments are based on the latest American Joint Committee on Cancer staging system for EC [5]. However, it is not difficult to find a significant difference in the prognosis of EC patients with similar TNM stages. Song et al. [9] found that even for EC patients with similar TNM stages, tumor length is still an independent factor for prognosis. In previous studies, many efforts have been made to find new clinicopathological factors to predict the prognosis of EC patients [56, 57].

Therefore, we conducted this meta-analysis to assess the prognostic significance of tumor length in EC patients. Tumor size has been used in staging assessment systems for lung and thyroid cancers [58, 59]. Many studies have been conducted on the relationship between tumor length and prognosis in other tumors [60, 61]. Our team published the protocol of this meta-analysis in September 2018 [62]. Later, Yang et al. [63] performed a meta-analysis of the relationship between prognosis and tumor length in patients undergoing radical resection of EC. On the basis of expanding the scope of the literature included, we excluded some literature with repeated data and included literature on the prognosis and tumor length of EC patients with non-surgical treatment. At the same time, the sample size and pathological types were expanded, and the guiding value of tumor length for the choice of treatment in different stages was discussed. In fact, in the American Joint Committee on Cancer (AJCC) TNM staging system in 1983, tumor length was used as an indicator to evaluate T staging [64, 65]. Although it was replaced in the 1987 version by depth of invasion of the esophageal wall, tumor length is still considered by many researchers to be a factor related to the extent of the peripheral invasion of the tumor. Our overall pooled results demonstrated that long tumors had a negative effect on OS, DFS, PFS, CSS and DSS in EC patients. Moreover, our subgroup analysis and sensitivity analysis results showed that our pooled HRs were stable and reliable. However, in the US category, the pooled HR was 1.457 (95% CI: 0.851–2.495, *I*^2^ = 84.10%, *ph* < .001) [6, 50–55]. This result suggests that the tumor length of EC has different effects on OS in different races and regions. In the adenocarcinoma group and the mixed group, the pooled HRs were 1.616 (95% CI: 0.950–2.748, *I*^2^ = 67.90%, *ph* < .05) [6, 22, 52] and 1.128 (95% CI: 0.895–1.421, *I*^2^ = 78.50%, *ph* < .01) [21, 44, 50, 54, 55]. This finding suggests that the prognostic significance of tumor length may be limited for other non-squamous carcinomas of the esophagus. From the results of the literature we included, the research on the prognosis and tumor length of advanced EC patients treated with non-surgical treatment is actually very limited. This may be because advanced patients cannot obtain direct pathological specimens through surgery, and the obstruction symptoms of advanced patients are often very severe, so the endoscopic measurement of tumors is also very difficult. We found that two of the three articles in which patients were only treated with CRT suggested that there was no significant correlation between tumor length and prognosis [26, 48]. In studies of CRT only, the included patients had a later TNM stage; for advanced patients, the systemic progression of the disease is rapid, and OS is poor, so the effect of tumor length on prognosis may also be limited. This point of view can be corroborated in studies with > 60% TNM stage III and IV EC. The pooled HR was 1.371 (95% CI: 0.968–1.941, I^2^ = 81.1%, *ph* < .001) [8, 25–27, 33, 50]. Based on the above findings, we believe that tumor length may be of greater value in the prognosis of SCC and early EC. We may regard tumor length as an independent factor to evaluate prognosis and as a further subdivision standard only for early EC patients so that more individualized treatment measures that patients can benefit from can be selected. Yamamoto et al. [29] considered that whether the length of the tumor is less than 5 cm and the circumferential spread is less than two-thirds can be used as the criteria for endoscopic resection of mucosal carcinoma; moreover, whether neoadjuvant therapy can cause some patients to be reconsidered for included based on this standard also reflects the importance of tumor length as a selection index. For the choice of surgical method, in addition to the location of the tumor, the length of the tumor is also of great significance. For some patients with tumors longer than 5 cm, even if the location is lower, McKeown surgery may be needed to ensure R0 resection rather than Ivor Lewis surgery. However, how can the tumor length be systematically taken as the standard to further subdivide EC patients with different stages and pathological types? Further detailed, large sample size case-control studies or large data sets may be needed.

There were several limitations in this meta-analysis that need to be addressed. First, our study only included research published in English, which may cause publication bias in this study. Second, the number of articles describing tumor length and DSS/PFS/DSS/CSS was insufficient, and the reference value was limited. Third, there were no corresponding data to analyse the relationship between tumor length and prognosis in different tumor locations. More importantly, there were many confounding factors, such as different resources of patients, pathological types, treatment strategies, durations of follow-up, sample sizes, proportions of lymph node metastasis, TNM stages and cut-off values of tumor length, which will increase the heterogeneity of this study. Although we conducted subgroup analyses and sensitivity analyses on the extracted data, these analyses could not fully explain the heterogeneity. Significant heterogeneity may affect the reference value of the meta-analysis results. We also assessed Begg’s funnel plot and performed Egger’s test to evaluate publication bias, and the results indicated that 12 articles on the relationship between tumor length and OS in EC have not been published. Though we adjusted the pooled result with the trim-and-fill method, the existence of significant publication bias suggests that we may have deficiencies in the selection and inclusion of literature. All of these limitations may hinder the application of the results in clinical work. More studies and larger sample size meta-analyses are needed to correct these limitations and improve the accuracy of the results.

## Conclusion

The tumor length of EC is widely measured in clinical work, but it is not often used as an indicator to evaluate prognosis and select treatment. Our results show that long tumors are related to poor survival outcomes in EC and can be considered an effective prognostic factor. This index can be used for the risk stratification of patients and is a promising prognostic index for clinical decision-making regarding EC treatment, especially for early-stage patients. However, due to the limitations listed above and the lack of more detailed case-control studies, more well-designed and large-scale studies are needed to confirm this conclusion in the future.

## Data Availability

The datasets that support the conclusions of this study are openly available in PubMed, Embase, and Web of Science.

## Abbreviations

EC: Esophageal cancer
HR: Hazard ratio
CI: Confidence interval
OS: Overall survival
DFS: Disease-free survival
PFS: Progression-free survival
DSS: Disease-specific survival
CSS: Cancer-specific survival
NOS: Newcastle-Ottawa quality assessment scale
SCC: Squamous cell carcinoma
AC: Adenocarcinoma
LN: Lymph node
NR: Not reported
